# Linking Changes to Intraspecific Trait Diversity to Community Functional Diversity and Biomass in Response to Snow and Nitrogen Addition Within an Inner Mongolian Grassland

**DOI:** 10.3389/fpls.2017.00339

**Published:** 2017-03-14

**Authors:** Wei Mao, Andrew J. Felton, Tonghui Zhang

**Affiliations:** ^1^Northwest Institute of Eco-Environment and Resource, Chinese Academy of SciencesLanzhou, China; ^2^Department of Biology and Graduate Degree Program in Ecology, Colorado State University, Fort CollinsCO, USA

**Keywords:** life history, community assembly, functional diversity, intraspecific variance, trait overlap

## Abstract

In recent years, both the intraspecific and interspecific functional diversity (FD) of plant communities have been studied with new approaches to improve an understanding about the mechanisms underlying plant species coexistence. Yet, little is known about how global change drivers will impact intraspecific FD and trait overlap among species, and in particular how this may scale to impacts on community level FD and ecosystem functioning. To address this uncertainty, we assessed the direct and indirect responses of specific leaf area (SLA) among both dominant annual and subordinate perennial species to the independent and interactive effects of nitrogen and snow addition within the Inner Mongnolian steppe. More specifically, we investigated the consequences for these responses on plant community FD, trait overlap and biomass. Nitrogen addition increased the biomass of the dominant annual species and as a result increased total community biomass. This occurred despite concurrent decreases in the biomass of subordinate perennial species. Nitrogen addition also increased intraspecific FD and trait overlap of both annual species and perennial species, and consequently increased the degree of trait overlap in SLA at the community level. However, snow addition did not significantly impact intraspecific FD and trait overlap of SLA for perennial species, but increased intraspecific FD and trait overlap of annual species, of which scaled to changes in community level FD. We found that the responses of the dominant annual species to nitrogen and snow additions were generally more sensitive than the subordinate perennial species within the inner Mongolian grassland communities of our study. As a consequence of this sensitivity, the responses of the dominant species largely drove impacts to community FD, trait overlap and community biomass. In total, our study demonstrates that the responses of dominant species in a community to environmental change may drive the initial trajectories of change to community FD and functioning.

## Introduction

Functional diversity (FD) can be a strong predictor of ecosystem processes (Albert et al., 2010; Jung et al., 2014; Lamanna et al., 2014), and has allowed ecologists to scale up the functional traits of individuals to the community level (de Bello et al., 2013; Kazakou et al., 2014). Previous findings suggest that communities with large interspecific FD are more likely to have species with traits adapted to novel environmental conditions, of which may facilitate rapid changes in the composition of community level traits through species turnover (Kraft et al., 2007; Weiher et al., 2011). As for intraspecific trait variability (ITV), previous research has typically assumed that ITV is small compared to interspecific variation (Kraft et al., 2008). However, recent evidence suggests that ITV may account for as much as 25% of the total trait variation on average within communities at the global scale (Siefert and Ritchie, 2016). Moreover, recent studies have highlighted the notion that ITV is likely to be important for contributing insight toward rules governing plant community assembly and ecosystem functioning (Violle et al., 2012; Auger and Shipley, 2013; Hulshof et al., 2013; Siefert et al., 2015).

For quantitative assessments of ITV, Violle et al. (2012) provided a framework for how to calculate internal filtering with using the T_IP/IC_ metric. T_IP/IC_ is the ratio of variation of trait values among individuals within populations (IP) to variation of trait values among individuals (IC) in the community, and thus is a strong indicator for assessing trait overlap between species for a given level of interspecific FD (Violle et al., 2012). Communities with high T_IP/IC_ indicate that species within the community have more similar traits, and vice versa. This assessment is also consistent with niche-based model approaches. As in the niche-based model, the dissimilarity or similarity within and between coexisting species can provide insight as to how the available resources are partitioned among species within the community (Weiher and Keddy, 1995; Cornwell and Ackerly, 2009; Mason et al., 2011).

For plant communities with highly uneven species abundances, the ITV of dominant species within the community may have a significant effect on community FD due to such species occupying larger niche space (Johnson et al., 2015). According to the ‘mass ratio hypothesis’ (Grime, 1998), the extent to which a trait of a plant species impacts ecosystem functioning is posited to be related to the contribution of a species to community composition and/or productivity. The underlying assumption of this hypothesis is that weighting traits by species abundance will improve the scaling of individual responses to community and ecosystem processes (Grime, 1998; Lavorel and Garnier, 2002). As a consequence, the function of an ecosystem likely depends to a great extent on the functional traits of the dominant species or functional groups within communities (Breza et al., 2012). Therefore, the intraspecific variation of dominant species may largely determine community trait space and the ability to obtain resources (Johnson et al., 2015), and may also impact other properties such as community biomass (Li et al., 2016). For example, dominant species with higher specific leaf area (SLA), photosynthetic efficiency, and thus resource acquisition have been shown to produce higher leaf biomass that directly scales to the community level, leading to a strong association between the dominant species traits and SLA at community scale, of which may further scale to community productivity (Li et al., 2016). As such, environmentally driven changes to key traits such as SLA may very well scale up to changes to the community and ecosystem level.

Interannual fluctuations of snow have increased due to increases in climate variability associated with global climate change, while anthropogenic nitrogen deposition has increased dramatically since the industrial revolution (Galloway et al., 2004; Bobbink et al., 2010; Liu et al., 2013). Anthropogenic nitrogen deposition includes the input of reactive nitrogen from the atmosphere to the biosphere from such sources as gasses, dry deposition and in precipitation as wet deposition. Increased nitrogen deposition will impact biodiversity at the global scale (Sala et al., 2000), and thus may also impact both ITV and FD within communities, of which can be represented by the variation in T_IP/IC_.

Intraspecific trait variability has been demonstrated to mediate the effects of climatic changes, such as extreme drought, on subalpine grassland communities (Jung et al., 2014), yet the dynamics of ITV may differ among species with different life histories. More specifically, annual species and perennial species often exhibit different patterns of responses to environmental change, which has been shown to impact species turnover and community productivity (Mao et al., 2012). For annual species, nitrogen addition has been observed to reduce the proportion of belowground biomass and increase aboveground biomass (Cai et al., 2005; Qi et al., 2011; Yan et al., 2013), while only significantly increasing the belowground biomass of perennial grasses (Song et al., 2012). Further, in low nutrient habitats annual species have been reported to have higher reproductive outputs than perennial species (Ploschuk et al., 2005).

Snow is a potential source of water via soil moisture and thus is critical for plant ecophysiological processes, such as biomass production (Wipf, 2010). Previous studies have shown that increased snow may be more important for perennials, and thus increased snowfall in winter could lead to changes in species composition due to greater benefits to perennial species (Chen et al., 2008; Aerts et al., 2009; Liu et al., 2011). For example, in the alpine meadow of the Qinghai–Tibet Plateau, increased snowfall increased the aboveground biomass of perennial grasses, yet had little effect on annual species (Shen et al., 2002). Although there is some evidence to suggest that snow and nitrogen deposition have interactive ecological effects (Zatko et al., 2016), few studies have analyzed the interaction between long-term, continuous nitrogen addition and interannual snow addition. Moreover, although the effects of nitrogen on intraspecific FD and interspecific FD have been previously documented (Koerner et al., 2016; Siefert and Ritchie, 2016), few studies have examined the effects on both intraspecific and interspecific FD, and in particular the role of dominant versus subordinate species responses in driving impacts at the community level. To address these uncertainties, we exposed a grassland ecosystem to multiple years of increased nitrogen and snow additions. The research site of this study is located in northern Inner Mongolia, which has come to be dominated by annual species due to land degradation (Mao et al., 2012). Therefore, we posited that changes to the ITV and/or FD of annual species may largely determine impacts at the community level. In this area, severe land desertification and subsequent wind erosion leads to loss of clay and silt particles, resulting in soil infertility and coarsening, and thus decreased soil water holding capacity and soil inorganic nitrogen availability (Zhou et al., 2008). Such dynamics make these already drought-prone and nutrient-poor plant communities increasingly subject to water and nutrient limitation.

Nitrogen addition may favor species that allocate more biomass to aboveground carbon assimilation organs (Bai et al., 2010; Koerner et al., 2016). However, the responses of plant species to nitrogen deposition is highly variable (Diekmann and Falkengren-Grerup, 2002). However, the effect of nitrogen addition on annual plant has been observed to be stronger than perennial species, with snow more strongly impacting perennial species (Wipf, 2010; Lü et al., 2014). Thus, if a community is exposed to both of these drivers, there will likely be trade-offs between snow and nitrogen effects on species performance within the community, particularly if the community is comprised of both perennial and annual species. In accordance with prior findings, we hypothesized that snow addition would significantly affect the intraspecific variation and traits of overlap of perennial species. However, we posited that nitrogen addition would increase the intraspecific and traits overlap of annual species in the community. This hypothesis lead to three key predictions:

(1) Nitrogen addition will more strongly affects annual plants via increased ITV, leading to higher T_IP/IC_.
(2) Snow addition will more strongly affected perennial plants via increased ITV and thus T_IP/IC_, yet the effect will disappear after cessation of snow addition.
(3) Changes in the ITV of the dominant annual species will more strongly scale to effects on community ITV and biomass than changes to the ITV of subordinate annual plants.

## Materials and Methods

### Experimental Design

The nitrogen and snow addition experiment was established in the Horqin Sandy Grassland of eastern Inner Mongolia, China (42°55′N, 120°42′E) on 5 October 2009 and ran through 10 October 2014. The total soil nitrogen content in the top 0–30 cm ranged from 0.057 to 0.199 (%), and the soil bulk density ranged from approximately 1.29–1.59 g cm^-3^ (Mao et al., 2012). Soil nitrogen and snow were treated as limiting factors that reflect both stressed and unstressed habitats depending on their availability. For the soil nitrogen, stressed conditions represented ambient conditions due to the characteristically low nutrient levels (represented by N_0_). For the unstressed condition, we added extra 536 g (equal to 100 kg ha y^-1^) of (NH_2_)_2_CO to each plot to represent high nutrient levels (represented by N_1_). For snow, control plots received no additional snow (represented by W_0_). For the high snow level, we added snow equivalent to 100 mm precipitation (weight conversion) applied during the winter (represented by W_1_).

Nitrogen was added each May from 2009 to 2014, and snow was added in the winter from December 2009 to April 2010, from December 2010 to April 2011, and from December 2011 to April 2012. Snow was collected outside of the experimental plots during snowfall in winter and added to the each W_1_ plots evenly. The snow added was equal to 30% of the average annual precipitation during the growing season from the last 20 years (Mao et al., 2012).

We used a randomized complete block design, consisting of 24 10 m × 10 m plots. We employed two nitrogen and two snow treatments, as well as their interactions. The four experimental treatments are as follows: (1) control (N_0_W_0_), (2) high nitrogen and low snow (N_1_W_0_), (3) high nitrogen and high snow (N_1_W_1_), and (4) low nitrogen and high snow (N_0_W_1_). Each treatment was replicated six times.

### Leaf Traits and Biomass Measurements

Vegetation surveys were carried out during peak biomass accumulation in August from 2010 to 2014. In order to prevent edge effects, the quadrats were set at least 1 m from the edge, with the size of each sampling plot 1 m × 1 m. Species richness, abundance and the e were measured in each plot. Aboveground biomass was clipped, dried at 65°C and weighed to the nearest 0.01 g. Root-type and cluster perennial species, like *Pennisetum purpureum* and *Cleistogenes squarrosa*, were used to calculate the number of ramets. All communities were located in a grazing-free enclosure.

Leaf samples were collected in August 2010–2014 from the same plots used to assess SLA (kg m^-2^). We randomly chose three individuals of the selected plants from the six plots of each treatment for leaf functional traits analysis. A single fully expanded mature leaf was selected from three individuals of each species and used to determine leaf area and dry mass (drying for 48 h at 65°C) (Cornelissen et al., 2003; Perez-Harguindeguy et al., 2013). Selected species were determined to be dominant based on their abundance by weight within each given community. The percent abundance of dominant annual species was higher than 80% of the community among all plots.

We selected SLA at the species level to describe the total FD at the community level. We chose this trait because it is directly related to plant competitive ability with respect to different levels of nitrogen availability, and reflects trade-offs between tolerances of environmental stressors (e.g., nutrient or climatic) (Lepš et al., 2011). High SLA also indicates a fast rate of resource uptake (Wright et al., 2004; Laughlin, 2014), and thus increases in SLA will likely enhance plant-atmosphere gas exchange (Lepš et al., 2011; Koerner et al., 2016).

### Functional Diversity Indices

The total community FD, within species FD, and between-species FD were quantified for each treatment (utilizing R code by de Bello et al., 2011). This method can be used with multi and single trait approaches (Albert et al., 2010; de Bello et al., 2011). The contribution of intraspecific variability to community variability was assessed via T_IP/IC,_ which is the ratio of within-species (IP) variance to total community variance (IC) (Violle et al., 2012). All FD was calculated on the basis of three groups of species data; the first set of data was based on all species in each community. The second data set was based only on the annual plants that were determined as dominant in the community. The third data set was based only on all perennial species that were determined as subordinate in the community (Mao et al., 2012). A null modeling method was used to analyze the trait overlap for each community within the four different treatments.

To make sure that the observed pattern of T_IP/IC_ responses to different environmental variables was non-random (Mason et al., 2011; Kumordzi et al., 2015a), trait overlap (i.e., T_IP/ICexpected_) was calculated by randomizing SLA values between all individuals in the given community. We utilized the equation T_IP/IC_SES = [T_IP/ICobserved_ – mean (T_IP/ICexpected_)]/sd (T_IP/ICexpected_) (for further details see Kumordzi et al., 2015a). We then developed a large matrix that takes into account both species relative abundance and richness within the community.

### Statistical Analyses

We analyzed the effect of nitrogen and snow on biomass of annual and biomass of perennial species using analysis of variance (ANOVA). We also analyzed the intraspecific FD, interspecific FD and total FD at the community scale (de Bello et al., 2011). A null model was used to analyze the significance (*p* < 0.05) of intraspecific FD, interspecific FD and total FD at the community scale, annual species scale and perennial species scale, and was calculated as the difference between the observed and expected values (Kumordzi et al., 2015a). We used both R statistical software (R Development Core Team, 2014) and SPSS (version 19.0; SPSS Inc., Chicago, IL, USA) to analyze the data.

## Results

**FIGURE 1:**
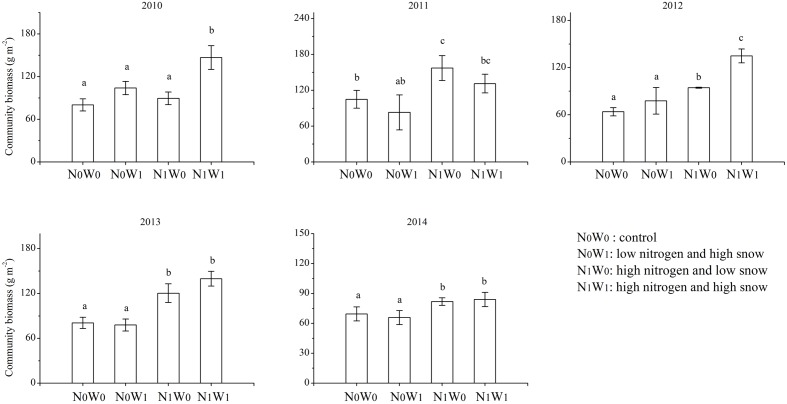
**The productivity of 2010–2014 at community scale.** Significance are indicated by different letters (*P* < 0.05).

**FIGURE 2:**
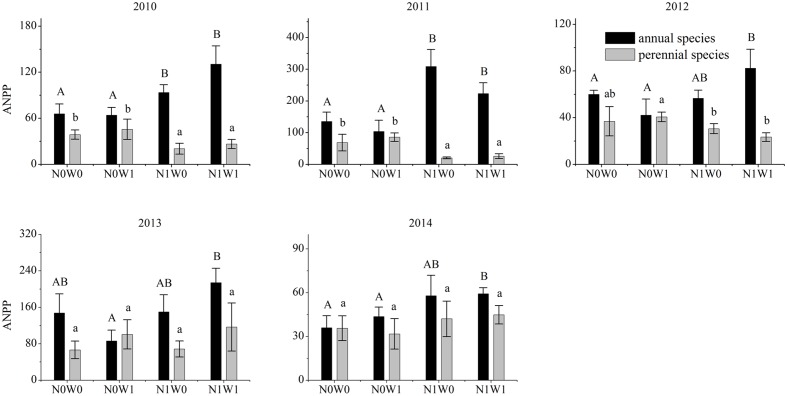
**ANPP of annual species and perennial species of 2010–2014.** Significance are indicated by different same type letters (*P* < 0.05).

Nitrogen addition increased community biomass, the biomass of annual species, yet reduced the biomass of perennial species (**Figures 1**, **2**). Compared with the control treatment, community biomass increased by 11.5, 49.8, 32.4, 49.4, and 17.7% after adding nitrogen in 2010, 2011, 2012, 2013, and 2014, respectively (**Figure 1**). In 2011, nitrogen addition increased annual species’ biomass by 128%. Surprisingly, the main effect of snow addition had no significant effect on biomass at the community, annual species or perennial species level (**Figure 2**). The interaction of nitrogen addition and snow addition significantly increased community biomass and the biomass of annual species. For example, in 2010, the interaction of nitrogen addition and snow addition increased 43.3% biomass of annual species, compared to control treatment (**Figure 2**).

One year after cessation of snow addition, there was still an interaction between nitrogen and snow additions. In 2013 the biomass of annual species in N_1_W_1_ treatment was 45.6% higher than snow additions alone. In 2014, nitrogen addition and the interaction of nitrogen and snow addition increased the biomass of annual species by 61.3 and 64.9%, respectively, compared to the control treatment (**Figure 2**). In 2010, 2011, and 2012, the biomass of perennial species at the nitrogen addition treatment decreased by 89.1, 235.9, and 20.6%, respectively, as compared with the control treatment. However, this trend began to weaken in the fourth year of nitrogen addition, and consequently nitrogen addition did not significantly reduce the biomass of perennial species in 2013.

**FIGURE 3:**
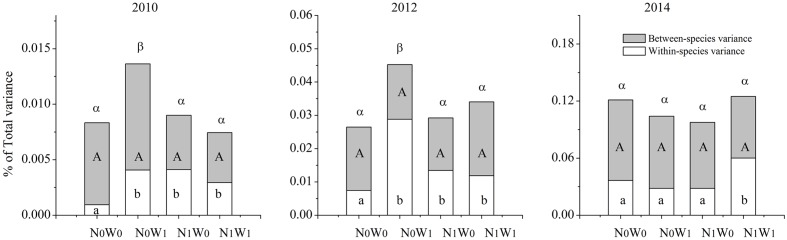
**Partitioning of the total functional diversity at community in to intraspecific functional diversity (transparent) and interspecific functional diversity (light gray) for SLA.** Significance are indicated by different same type letters (*P* < 0.05).

**FIGURE 4:**
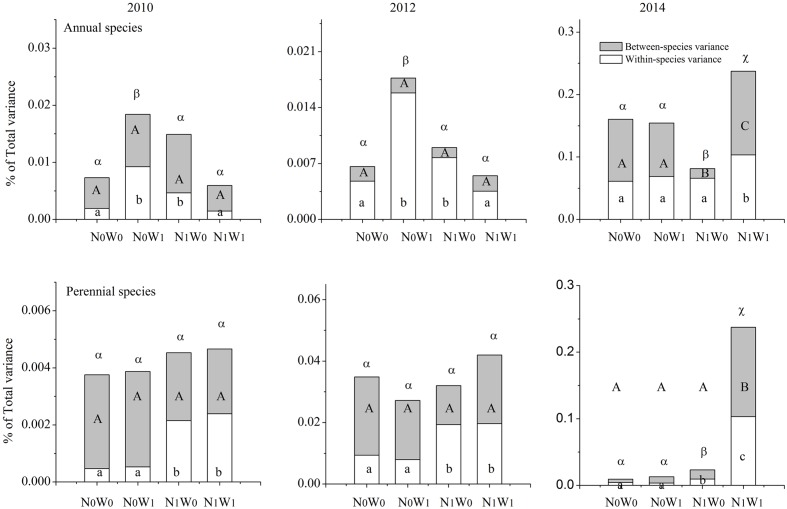
**Partitioning of the total functional diversity of annual species and perennial species in to intraspecific functional diversity (transparent) and interspecific functional diversity (light gray) for SLA.** Significance are indicated by different same type letters (*P* < 0.05).

We chose the first year of snow addition in 2010, and the last year of snow addition in 2012, and the second year following cessation of snow addition in 2014 to analyze the SLA of all coexisting species in the community. The results show that the effects of nitrogen addition, snow addition, and their interaction on intraspecific FD are significant, while the effects on the interspecific FD and total FD were comparatively weak (**Figure 3**). However, these effects differed among annual and perennial species. For example, snow addition in 2010 and 2012 increased intraspecific variation in annual species yet had no significant effect on perennials (**Figure 4**). Conversely, the interaction of nitrogen and snow addition dramatically affected the intraspecific variation of perennial species, yet not annual species in 2010 and 2012 (**Figure 4**). Despite these results, the nitrogen addition, snow addition and the interaction of nitrogen and snow ultimately had little effect on interspecific variation. However, in 2014 N addition did significantly impact the interspecific variation of annual species, while the interaction of nitrogen addition and snow addition affected the perennial interspecific variation significantly.

The trends of T_IP/IC_ after nitrogen addition and snow addition treatment were similar to that of intraspecific FD and total FD. The effects of nitrogen addition on T_IP/IC_ of the community, T_IP/IC_ of annual species and T_IP/IC_ of perennial species were significant (**Table 1**). After snow addition, the T_IP/IC_ of annual species increased markedly despite no impacts to biomass. However, T_IP/IC_ of annual species returned to near control levels after cessation of snow additions. Snow addition had no significant effect on the T_IP/IC_ of perennial species. Although the trend of biomass and community level ITV had no significant correlations, changes to the ITV and biomass of dominant plants were consistent with each other.

**Table 1:** The trait overlap of observed community, annual species and perennial species.

	Treatments∖T_IP/IC_	2010	2012	2014
Community	N_0_W_0_	0.240	0.342	0.268
	N_0_W_1_	**0.445^∗^**	0.468	0.382
	N_1_W_0_	**0.427^∗^**	**0.680^∗^**	**0.451^∗^**
	N_1_W_1_	**0.442^∗^**	0.384	**0.442^∗^**
Annual species	N_0_W_0_	0.272	0.467	0.420
	N_0_W_1_	**0.521^∗^**	**0.652^∗^**	0.309
	N_1_W_0_	**0.405^∗^**	**0.675^∗^**	**0.534^∗^**
	N_1_W_1_	0.321	**0.564^∗^**	0.406
Perennial species	N_0_W_0_	0.340	0.366	0.462
	N_0_W_1_	0.251	0.276	0.309
	N_1_W_0_	**0.686^∗^**	**0.465^∗^**	**0.534^∗^**
	N_1_W_1_	**0.632^∗^**	**0.451^∗^**	0.406


*All the data is log-transformed; Significance are indicated by star symbol (^∗^*P* < 0.05).*

## Discussion

### The Effect of Nitrogen Addition on Intraspecific FD and Interspecific FD

We observed shifts in the trait overlap between annual species and perennial species in response to key global change drivers of the study region, of which further underscores the potential role of niche packing as a central mechanism for the coexistence of plant species (Kraft et al., 2008; Mason et al., 2011; Le Bagousse-Pinguet et al., 2015; Fajardo and Siefert, 2016). Due to land desertification and subsequent wind erosion, the available soil nitrogen in this grassland is very low (Zhou et al., 2008). As a result of this limitation, the majority of the dominant species in this grassland are annual species, which are also sensitive to changes in nitrogen availability (Bai et al., 2010; Mao et al., 2012).

Indeed, in the plant communities of our study, the dominant annual species were highly sensitive to nitrogen addition (Mao et al., 2012). We observed that increased species trait overlap within the community due to nitrogen addition was mainly caused by increases to the ITV of the dominant annual species. In this nutrition-poor habitat, nitrogen addition alleviates nutrient limitation and thus alters the role of environmental filtering as a mechanism of community assembly (Chapin et al., 1986; den Haan et al., 2016), as nitrophilic plants may show more advantages as nitrogen levels increase (Menge et al., 2015). Moreover, coexisting species are likely to exhibit more nitrophilic traits in the community after nitrogen addition, and as a result trait overlap will likely increase.

The responses of the subordinate perennial species to nitrogen addition was different than annual species (**Supplementary Figure S1**). It is likely that light competition increased after nitrogen addition (Hautier et al., 2009; Okubo et al., 2012). This may then increase the upper limit of the SLA range, and further increase ITV via greater trait overlap among individuals. However, biomass of the dominant perennial species increased quickly after nitrogen addition and thus may have ultimately heightened competitive exclusion of perennial species in the community. This dynamic may operate to reduce trait overlap. The trait overlap of perennial species is likely the result of the trade-off between the overlap increase caused by nitrogen addition and the overlap decrease caused by the increase in the intensity of competition. The trait overlap of perennial species increased after nitrogen addition (Le Bagousse-Pinguet et al., 2015), which indicates that environmental filtering played a more obvious role in driving increased trait overlap as compared to interspecific competition (Kumordzi et al., 2015a). These results are consistent with hypothesis i.

We did not find a detectable decrease in interspecific FD variation after nitrogen addition. This result contradicts with the prediction that nitrogen addition will impact species composition, and thus potentially increase the functional trait diversity at community scale (Weiher et al., 2011; Siefert and Ritchie, 2016) by extending the niche space of species in the community at the interspecific level (Kumordzi et al., 2015a). There may be two reasons for this result. First, the dominant species in our sandy grassland communities are annual forb species. The intraspecific variability of these species increased sharply after nitrogen addition, which may have obscured the impacts caused by interspecific FD variation. For example, in Qinghai Tibet the dominant species maximize resource use after nitrogen addition through intraspecific variability in SLA to occupy greater niche space, thus allowing more nitrogenous plants to coexist (Li et al., 2016) and leading to higher T_IP/IC_ in the community. Second, the species richness is very low in our study area. In species poor communities, the intraspecific variation is typically much higher than species-rich communities (Mao et al., 2012), while in comparison the interspecific variation is relatively small.

### The Effect of Snow Addition on Intraspecific FD and Interspecific FD

We found that snow did not change the intraspecific FD variation and trait overlap of the subordinate perennial species, which is not consistent with hypothesis ii. However the dominant annual species in the community increased their intraspecific FD variation and trait overlap after snow addition. The reason for the weak effects of snow on perennial species may be that, firstly, snow addition in this study did not directly affect the growth of perennials. The increase of snow in winter increases the thickness of snow, soil temperature and soil water supply in early spring, and thus affects plant growth and biomass allocation (Chen et al., 2008; Wipf, 2010). However, in the study area of Inner Mongolia, strong continuous winds blow accumulated snow rapidly, while the sandy soil also has poor water holding capacity (Yao et al., 2013). As a consequence, water produced after snowmelt may migrate to deep soil layers very quickly, where plant roots may not be able to fully access this resource.

Second, although snow addition slightly increased available soil available nutrients, this did not impact perennial species. This may be due to the fact that the perennial species in our study were mostly clonal plants, and that redistribution of available nutrients among individual clones could reduce the importance of small-scale spatial heterogeneity for FD and community assembly (Gundale et al., 2011). Patterns of snowfall are likely to change within the Inner Mongolian region, with these changes are less certain as compared to temperature change. One key prediction is that the amount of snowfall will increase each time, yet that the interval between events will also increase (IPCC, 2013). In this snowfall change scenarios, different functional groups of plants show different response strategies. Thus it may be the case that the response of annuals to snow changes is more pronounced in arid and semiarid regions, while perennial responses may comparatively insensitive to snow changes (Mao et al., 2012).

The change of trait overlap of annual species was more obvious after snow addition, and the variation in this pattern was similar to the changes at the community scale. This result is consistent with the ‘mass ratio hypothesis,’ which posits that the function of the ecosystem is largely determined by the traits of the dominant species or functional groups (Grime, 1998; Johnson et al., 2015). Therefore, the ITV of SLA for the dominant annual species is likely to play a more important role in determining patterns of community level FD than that of subordinate species (Lavorel and Garnier, 2002; Mao et al., 2012; Volf et al., 2016). The different variance pattern of annual species and perennial species suggests that changes to the niche space occupied by the dominant species or functional group will likely affect niche packing at the community level (Breza et al., 2012; Li et al., 2016). These contrasting responses of intraspecific FD and interspecific FD between dominant and subordinate species also suggests that species niche packaging plays an important role in maintaining the FD of plant communities (Johnson et al., 2015).

Although the effects of snow addition alone on intraspecific FD and interspecific FD of sandy grassland ecosystems were weak, there was an interaction between snow addition and nitrogen addition. This interaction remained 1 or 2 years after cessation of snow addition. Even if the interaction between snow addition and nitrogen addition are mostly driven by the effect of nitrogen alone, increasing snowfall amount may lend insight to potential variability in the effects of nitrogen on the community (**Figure 4**). For instance, the interaction of snow addition and nitrogen addition on intraspecific variability are significant, while the effect on interspecific FD was weak. There were temporally different responses between annual species and perennial species in N_1_W_1_ treatment. For instance, the interaction of nitrogen and snow had no significant effect on intraspecific FD and interspecific FD of annual species in 2010 and 2012 when both nitrogen and snow were added, yet affected the intraspecific FD and interspecific FD of perennial species. This is clearly different from the addition of nitrogen alone.

### The Variation of T_IP/IC_ and Biomass

**FIGURE 5:**
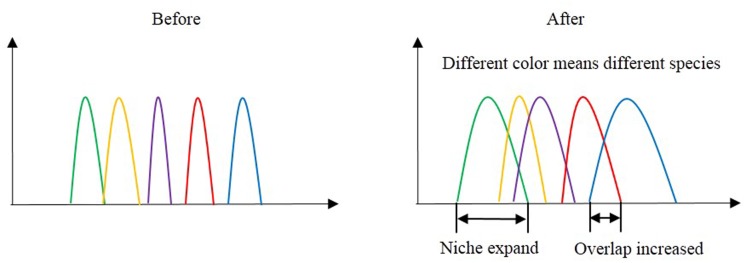
**Niche overlap before and after nitrogen addition and snow addition**.

The response of intraspecific variation, T_IP/IC_ and biomass of the dominant annual species to nitrogen addition were related to trends at the community level, and the response of the annual species differed from that of the subordinate perennial species (**Figure 5** and **Supplementary Figure S2**). This result supports our third hypothesis. Previous studies have shown that communities with higher intraspecific FD, potentially indicative of high phenotypic plasticity or genetic variation, may also have increased capacity to respond to environmental change through changes in traits within the same species (Grime, 2006). In this study, the biomass allocation of dominant species changed after nitrogen addition, and more nutrients were likely distributed to the leaves, which expanded the range of leaf traits and increased leaf, species and community biomass. Conversely, the effect of nitrogen addition on perennial plants was relatively weak and even negative (Mao et al., 2012). We also used standardized major axis tests to analyze the variation of annual species and perennial species after nitrogen addition. The results indicate that the resource utilization axis of the annual species shifted to the direction of nitrogen use after nitrogen addition, and that the community showed more nitrophilic functional traits (e.g., high SLA and leaf nitrogen content) as the ratio of leaf weight to total weight increased significantly (Mao et al., 2012). This effect in turn increased the biomass of the community, without significant changes in biomass allocation of perennial species after nitrogen addition.

The biomass of the community and biomass of annual species increased at nitrogen addition treatment and N_1_W_1_ treatment, yet interestingly the biomass of perennial species decreased significantly. This contrasting result suggests that different community components, such as dominant versus subordinate species, will likely have differential responses to environmental change drivers (Fargione and Tilman, 2006; Kumordzi et al., 2015b). Moreover, depending on a species role in the community, the response of a species to environmental change may or may not translate to detectable impacts at the community level. For example, community biomass was mainly affected by the biomass of dominant annual species, which showed the same trend with the T_IP/IC_ of annual plants, despite a negative trend with the changes of subordinate perennial plant biomass. This further suggests that changes in the functional traits and productivity of dominant and subordinate species may help to more accurately understand which component of the community has a greater impact on community assembly and potentially ecosystem processes.

## Conclusion

We analyzed the effects of nitrogen addition and snow addition on the intraspecific and interspecific variation of the community, annual species, and perennial species. We studied how changes to these environmental factors may influence coexistence, community FD and biomass. First, nitrogen addition increased the biomass, intraspecific variation and total FD of the annual plants, and eventually increased it’s T_IP/IC_. Nitrogen addition reduced the biomass of and intraspecific variation, and trait overlap of perennials, yet did not change total FD. Second, snow addition did not change the biomass of annual species, but increased intraspecific variation at the community scale by increasing the intraspecific variation of annuals. Snow addition also had no effect on the biomass, ITV and trait overlap of perennial plants. In total, the ITV of SLA of the dominant annual plant species, a trait related to plant resource acquisition capacity, partially informed changes to total community FD and biomass. Thus, global change-driven trait shifts among dominant species within plant communities, particularly if they are species-poor, are likely to underlie the initial changes to community FD and function.

## Author Contributions

WM carried out this research, collected the field data and draft the manuscript, carried out all data analysis. TZ conceived of the study, designed the study and AF helped draft the manuscript; All author gave final approval for publication.

## Conflict of Interest Statement

The authors declare that the research was conducted in the absence of any commercial or financial relationships that could be construed as a potential conflict of interest.

## Supplementary Material

**FIGURE S1 | The variance of richness, abundance and coverage between annual species and perennial species.**

**FIGURE S2 | The variance extent of intraspecific functional diversity and productivity.**

## References

[B1] Aerts R. Callaghan T. V. Dorrepaal E. Logtestijn R. S. P. V. Cornelissen J. H. C. (2009). Seasonal climate manipulations result in species-specific changes in leaf nutrient levels and isotopic composition in a sub-arctic bog. *Funct. Ecol.* 23 680–688. 10.1111/j.1365-2435.2009.01566.x

[B2] Albert C. H. Thuiller W. Yoccoz N. G. Soudant A. Boucher F. Saccone P. (2010). Intraspecific functional variability: extent, structure and sources of variation. *J. Ecol.* 98 604–613. 10.1111/j.1365-2745.2010.01651.x

[B3] Auger S. Shipley B. (2013). Inter-specific and intra-specific trait variation along short environmental gradients in an old-growth temperate forest. *J. Veg. Sci.* 24 419–428. 10.1111/j.1654-1103.2012.01473.x

[B4] Bai Y. Wu J. Clark C. M. Naeem S. Pan Q. Huang J. (2010). Tradeoffs and thresholds in the effects of nitrogen addition on biodiversity and ecosystem functioning: evidence from inner Mongolia Grasslands. *Glob. Chang Biol.* 16 358–372. 10.1111/j.1365-2486.2009.01950.x

[B5] Bobbink R. Hicks K. Galloway J. Spranger T. Alkemade R. Ashmore M. (2010). Global assessment of nitrogen deposition effects on terrestrial plant diversity: a synthesis. *Ecol. Appl.* 20 30–59. 10.1890/08-1140.1 20349829

[B6] Breza L. C. Souza L. Sanders N. J. Classen A. T. (2012). Within and between population variation in plant traits predicts ecosystem functions associated with a dominant plant species. *Ecol. Evol.* 2 1151–1161. 10.1002/ece3.223 22833791 PMC3402191

[B7] Cai X. Li Z. Chen Z. Wang Y. Wang S. Wang Y. (2005). The relationship between aboveground biomass and precipitation on *Stipa grandis* steppe in Inner Mongolia. *Acta Ecol. Sin.* 25 1657–1662.

[B8] Chapin I. I. I. F. S. Vitousek P. M. Cleve K. V. (1986). The nature of nutrient limitation in plant communities. *Am. Nat.* 127 48–58. 10.1086/284466

[B9] Chen W. N. Wu Y. Wu N. Luo P. (2008). Effect of snow-cover duration on plant species diversity of alpine meadows on the eastern Qinghai-Tibetan Plateau. *J. Mt. Sci.* 5 327–339. 10.1007/s11629-008-0182-0

[B10] Cornelissen J. H. C. Lavorel S. Garnier E. Diaz S. Buchmann N. Gurvich D. E. (2003). A handbook of protocols for standardised and easy measurement of plant functional traits worldwide. *Aust. J. Bot.* 51 335–380. 10.1071/bt02124

[B11] Cornwell W. K. Ackerly D. D. (2009). Community assembly and shifts in plant trait distributions across an environmental gradient in coastal California. *Ecol. Monogr.* 79 109–126. 10.1890/07-1134.1

[B12] de Bello F. Carmona C. P. Mason N. W. H. Sebastià M.-T. Lepš J. (2013). Which trait dissimilarity for functional diversity: traitmeans or trait overlap? *J. Veg. Sci.* 24 807–819. 10.1111/jvs.12008

[B13] de Bello F. Lavorel S. Albert C. H. Thuiller W. Grigulis K. Dolezal J. (2011). Quantifying the relevance of intraspecific trait variability for functional diversity. *Methods Ecol. Evol.* 2 163–174. 10.1111/j.2041-210X.2010.00071.x

[B14] den Haan J. Huisman J. Brocke H. J. Goehlich H. Latijnhouwers K. R. W. van Heeringen S. (2016). Nitrogen and phosphorus uptake rates of different species from a coral reef community after a nutrient pulse. *Sci. Rep.* 6:28821 10.1038/srep28821 PMC4926277 27353576

[B15] Diekmann M. Falkengren-Grerup U. (2002). Prediction of species response to atmospheric nitrogen deposition by means of ecological measures and life history traits. *J. Ecol.* 90 108–120. 10.1046/j.0022-0477.2001.00639.x

[B16] Fajardo A. Siefert A. (2016). Phenological variation of leaf functional traits within species. *Oecologia* 180 951–959. 10.1007/s00442-016-3545-1 26796408

[B17] Fargione J. Tilman D. (2006). Plant species traits and capacity for resource reduction predict yield and abundance under competition in nitrogen-limited grassland. *Funct. Ecol.* 20 533–540. 10.1111/j.1365-2435.2006.01116.x

[B18] Galloway J. N. Dentener F. J. Capone D. G. Boyer E. W. Howarth R. W. Seitzinger S. P. (2004). Nitrogen cycles: past. present, and future. *Biogeochemistry* 70 153–226. 10.1007/s10533-004-0370-0

[B19] Grime J. P. (1998). Benefits of plant diversity to ecosystems: immediate, filter and founder effects. *J. Ecol.* 86 902–910. 10.1046/j.1365-2745.1998.00306.x

[B20] Grime J. P. (2006). Trait convergence and trait divergence in herbaceous plant communities: mechanisms and consequences. *J. Veg. Sci.* 17 255–260. 10.1111/j.1654-1103.2006.tb02444.x

[B21] Gundale M. J. Deluca T. H. Nordin A. (2011). Bryophytes attenuate anthropogenic nitrogen inputs in boreal forests. *Glob. Chang Biol.* 17 2743–2753. 10.1111/j.1365-2486.2011.02407.x

[B22] Hautier Y. Niklaus P. A. Hector A. (2009). Competition for light causes plant biodiversity loss after eutrophication. *Science* 324 636–638. 10.1126/science.1169640 19407202

[B23] Hulshof C. M. Violle C. Spasojevic M. J. McGill B. Damschen E. Harrison S. (2013). Intra-specific and inter-specific variation in specific leaf area reveal the importance of abiotic and biotic drivers of species diversity across elevation and latitude. *J. Veg. Sci.* 24 921–931. 10.1111/jvs.12041

[B24] IPCC (2013). *Climate Change 2013: The Physical Science Basis. Contribution of Working Group I to the Fifth Assessment Report of the Intergovernmental Panel on Climate Change*. New York, NY: Cambridge University Press.

[B25] Johnson L. C. Olsen J. T. Tetreault H. DeLaCruz A. Bryant J. Morgan T. J. (2015). Intraspecific variation of a dominant grass and local adaptation in reciprocal garden communities along a US Great Plains’ precipitation gradient: implications for grassland restoration with climate change. *Evol. Appl.* 8 705–723. 10.1111/eva.12281 26240607 PMC4516422

[B26] Jung V. Albert C. H. Violle C. Kunstler G. Loucougaray G. Spiegelberger T. (2014). Intraspecific trait variability mediates the response of subalpine grassland communities to extreme drought events. *J. Ecol.* 102 45–53. 10.1111/1365-2745.12177

[B27] Kazakou E. Violle C. Roumet C. Navas M.-L. Vile D. Kattge J. (2014). Are trait-based species rankings consistent across data sets and spatial scales? *J. Veg. Sci.* 25 235–247. 10.1111/jvs.12066

[B28] Koerner S. E. Avolio M. L. La Pierre K. J. Wilcox K. R. Smith M. D. Collins S. L. (2016). Nutrient additions cause divergence of tallgrass prairie plant communities resulting in loss of ecosystem stability. *J. Ecol.* 104 1478–1487. 10.1111/1365-2745.12610

[B29] Kraft N. J. B. Cornwell W. K. Webb C. O. Ackerly D. D. (2007). Trait evolution, community assembly, and the phylogenetic structure of ecological communities. *Am. Nat.* 170 271–283. 10.1086/519400 17874377

[B30] Kraft N. J. B. Valencia R. Ackerly D. D. (2008). Functional traits and niche-based tree community assembly in an amazonian forest. *Science* 322 580–582. 10.1126/science.1160662 18948539

[B31] Kumordzi B. B. de Bello F. Freschet G. T. Le Bagousse-Pinguet Y. Lepš J. Wardle D. A. (2015a). Linkage of plant trait space to successional age and species richness in boreal forest understorey vegetation. *J. Ecol.* 103 1610–1620. 10.1111/1365-2745.12458

[B32] Kumordzi B. B. Wardle D. A. Freschet G. T. (2015b). Plant assemblages do not respond homogenously to local variation in environmental conditions: functional responses differ with species identity and abundance. *J. Veg. Sci.* 26 32–45. 10.1111/jvs.12218

[B33] Lamanna C. Blonder B. Violle C. Kraft N. J. B. Sandel B. Šímová I. (2014). Functional trait space and the latitudinal diversity gradient. *Proc. Natl. Acad. Sci. U.S.A.* 111 13745–13750. 10.1073/pnas.1317722111 25225365 PMC4183280

[B34] Laughlin D. C. (2014). The intrinsic dimensionality of plant traits and its relevance to community assembly. *J. Ecol.* 102 186–193. 10.1111/1365-2745.12187

[B35] Lavorel S. Garnier E. (2002). Predicting changes in community composition and ecosystem functioning from plant traits: revisiting the Holy Grail. *Funct. Ecol.* 16 545–556. 10.1046/j.1365-2435.2002.00664.x

[B36] Le Bagousse-Pinguet Y. Börger L. Quero J.-L. García-Gómez M. Soriano S. Maestre F. T. (2015). Traits of neighbouring plants and space limitation determine intraspecific trait variability in semi-arid shrublands. *J. Ecol.* 103 1647–1657. 10.1111/1365-2745.12480

[B37] Lepš J. de Bello F. Šmilauer P. Doležal J. (2011). Community trait response to environment: disentangling species turnover vs intraspecific trait variability effects. *Ecography* 34 856–863. 10.1111/j.1600-0587.2010.06904.x

[B38] Li W. Zhao J. Epstein H. E. Jing G. Cheng J. Du G. (2016). Community-level trait responses and intra-specific trait variability play important roles in driving community productivity in an alpine meadow on the Tibetan Plateau. *J. Plant Ecol.* 10.1093/jpe/rtw069

[B39] Liu L. Wu Y. He Y. X. Wu N. Sun G. Zhang L. (2011). Effects of seasonal snow cover on soil nitrogen transformation in alpine ecosystem: a review. *Chin. J. Appl. Ecol.* 22 2193–2200. 22097387

[B40] Liu X. Zhang Y. Han W. Tang A. Shen J. Cui Z. (2013). Enhanced nitrogen deposition over China. *Nature* 494 459–462. 10.1038/nature11917 23426264

[B41] Lü X.-T. Dijkstra F. A. Kong D.-L. Wang Z.-W. Han X.-G. (2014). Plant nitrogen uptake drives responses of productivity to nitrogen and water addition in a grassland. *Sci. Rep.* 4 1–7. 10.1038/srep04817 PMC4001094 24769508

[B42] Mao W. Ginger A. Li Y. L. Zhang T. H. Zhao X. Y. Huang Y. X. (2012). Life history strategy influences biomass allocation in response to limiting nutrients and water in an arid system. *Pol. J. Ecol.* 60 381–389.

[B43] Mason N. W. H. de Bello F. Doležal J. Lepš J. (2011). Niche overlap reveals the effects of competition, disturbance and contrasting assembly processes in experimental grassland communities. *J. Ecol.* 99 788–796. 10.1111/j.1365-2745.2011.01801.x

[B44] Menge D. N. L. Wolf A. A. Funk J. L. (2015). Diversity of nitrogen fixation strategies in Mediterranean legumes. *Nature Plants* 1 15064 10.1038/nplants.2015.64 27250004

[B45] Okubo S. Tomatsu A. Parikesit P. Muhamad D. Harashina K. Takeuchi K. (2012). Leaf functional traits and functional diversity of multistoried agroforests in West Java, Indonesia. *Agric. Ecosyst. Environ.* 149 91–99. 10.1016/j.agee.2011.12.017

[B46] Perez-Harguindeguy N. Diaz S. Garnier E. Lavorel S. Poorter H. Jaureguiberry P. (2013). New handbook for standardised measurement of plant functional traits worldwide. *Aust. J. Bot.* 61 167–234. 10.1071/BT12225

[B47] Ploschuk E. Slafer G. Ravetta D. (2005). Reproductive allocation of biomass and nitrogen in annual and perennial Lesquerella crops. *Ann. Bot.* 96 127–135. 10.1093/aob/mci158 15863469 PMC4246817

[B48] Qi Y. Huang Y. Wang Y. Zhao J. Zhang J. (2011). Biomass and its allocation of four grassland species under different nitrogen levels. *Acta Ecol. Sin.* 31 5121–5129.

[B49] R Development Core Team (2014). *R: A Language and Environment for Statistical Computing*. Vienna: R Foundation for Statistical Computing.

[B50] Sala O. E. Chapin FS 3rd Armesto J. J. Berlow E. Bloomfield J. Dirzo R. (2000). Global biodiversity scenarios for the year 2100. *Science* 287 1770–1774. 10.1126/science.287.5459.1770 10710299

[B51] Shen Z. X. Zhou X. M. Chen Z. Z. Zhou H. K. (2002). Response of plant groups to simulated rainfall and nitrogen supply in alpine kobresia humilis meadow. *Acta Phytoecol. Sin.* 26 288–294. 10.3321/j.issn:1005-264X.2002.03.006

[B52] Siefert A. Ritchie M. E. (2016). Intraspecific trait variation drives functional responses of old-field plant communities to nutrient enrichment. *Oecologia* 181 245–255. 10.1007/s00442-016-3563-z 26826004

[B53] Siefert A. Violle C. Chalmandrier L. Albert C. H. Taudiere A. Fajardo A. (2015). A global meta-analysis of the relative extent of intraspecific trait variation in plant communities. *Ecol. Lett.* 18 1406–1419. 10.1111/ele.12508 26415616

[B54] Song L. Bao X. Liu X. Zhang F. (2012). Impact of nitrogen addition on plant community in a semi-arid temperate steppe in China. *J. Arid Land* 4 3–10. 10.3724/SP.J.1227.2012.00003

[B55] Violle C. Enquist B. J. McGill B. J. Jiang L. Albert C. H. Hulshof C. (2012). The return of the variance: intraspecific variability in community ecology. *Trends Ecol. Evol.* 27 244–252. 10.1016/j.tree.2011.11.014 22244797

[B56] Volf M. Redmond C. Albert ÁJ. Le Bagousse-Pinguet Y. Biella P. Götzenberger L. (2016). Effects of long- and short-term management on the functional structure of meadows through species turnover and intraspecific trait variability. *Oecologia* 180 941–950. 10.1007/s00442-016-3548-y 26837384

[B57] Weiher E. Freund D. Bunton T. Stefanski A. Lee T. Bentivenga S. (2011). Advances, challenges and a developing synthesis of ecological community assembly theory. *Philos. Trans. R. Soc. B Biol. Sci.* 366 2403–2413. 10.1098/rstb.2011.0056 PMC3130429 21768155

[B58] Weiher E. Keddy P. A. (1995). Assembly rules, null models, and trait dispersion : new questions from old patterns. *Oikos* 74 159–164. 10.2307/3545686

[B59] Wipf S. (2010). Phenology, growth, and fecundity of eight subarctic tundra species in response to snowmelt manipulations. *Plant Ecol.* 207 53–66. 10.1007/s11258-009-9653-9

[B60] Wright I. J. Reich P. B. Westoby M. Ackerly D. D. Baruch Z. Bongers F. (2004). The worldwide leaf economics spectrum. *Nature* 428 821–827. 10.1038/nature02403 15103368

[B61] Yan J. Liang C. Fu X. Wang W. Wang L. Jia C. (2013). The responses of annual plant traits to rainfall variation in steppe and desert regions. *Acta Prataculturae Sin.* 22 68–76.

[B62] Yao S. Zhang T. Zhao C. Liu X. (2013). Saturated hydraulic conductivity of soils in the Horqin Sand Land of Inner Mongolia, northern China. *Environ. Monit. Assess.* 185 6013–6021. 10.1007/s10661-012-3002-5 23179727

[B63] Zatko M. Geng L. Alexander B. Sofen E. Klein K. (2016). The impact of snow nitrate photolysis on boundary layer chemistry and the recycling and redistribution of reactive nitrogen across Antarctica and Greenland in a global chemical transport model. *Atmos. Chem. Phys.* 16 2819–2842. 10.5194/acp-16-2819-2016

[B64] Zhou R. L. Li Y. Q. Zhao H. L. Drake S. (2008). Desertification effects on C and N content of sandy soils under grassland in Horqin, northern China. *Geoderma* 145 370–375. 10.1016/j.geoderma.2008.04.003

