# Supplementary figures and images for: Linking Changes to Intraspecific Trait Diversity to Community Functional Diversity and Biomass in Response to Snow and Nitrogen Addition Within an Inner Mongolian Grassland
Source: Front Plant Sci. 2017 Mar 14;8:339. doi: 10.3389/fpls.2017.00339 (PMC5348515; doi:10.3389/fpls.2017.00339)

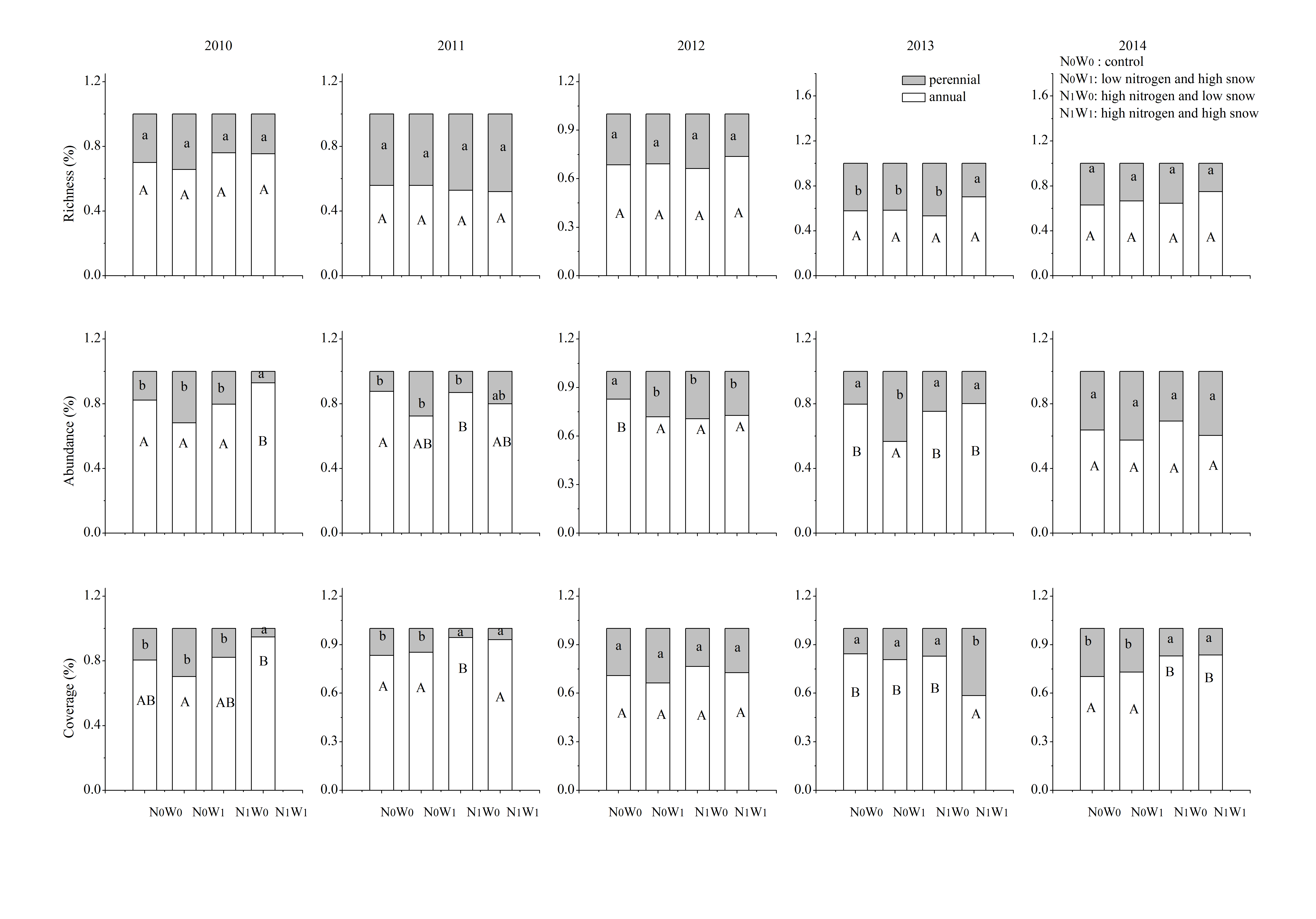

Supplement: Supplementary file 1 [file Image_1.JPEG]

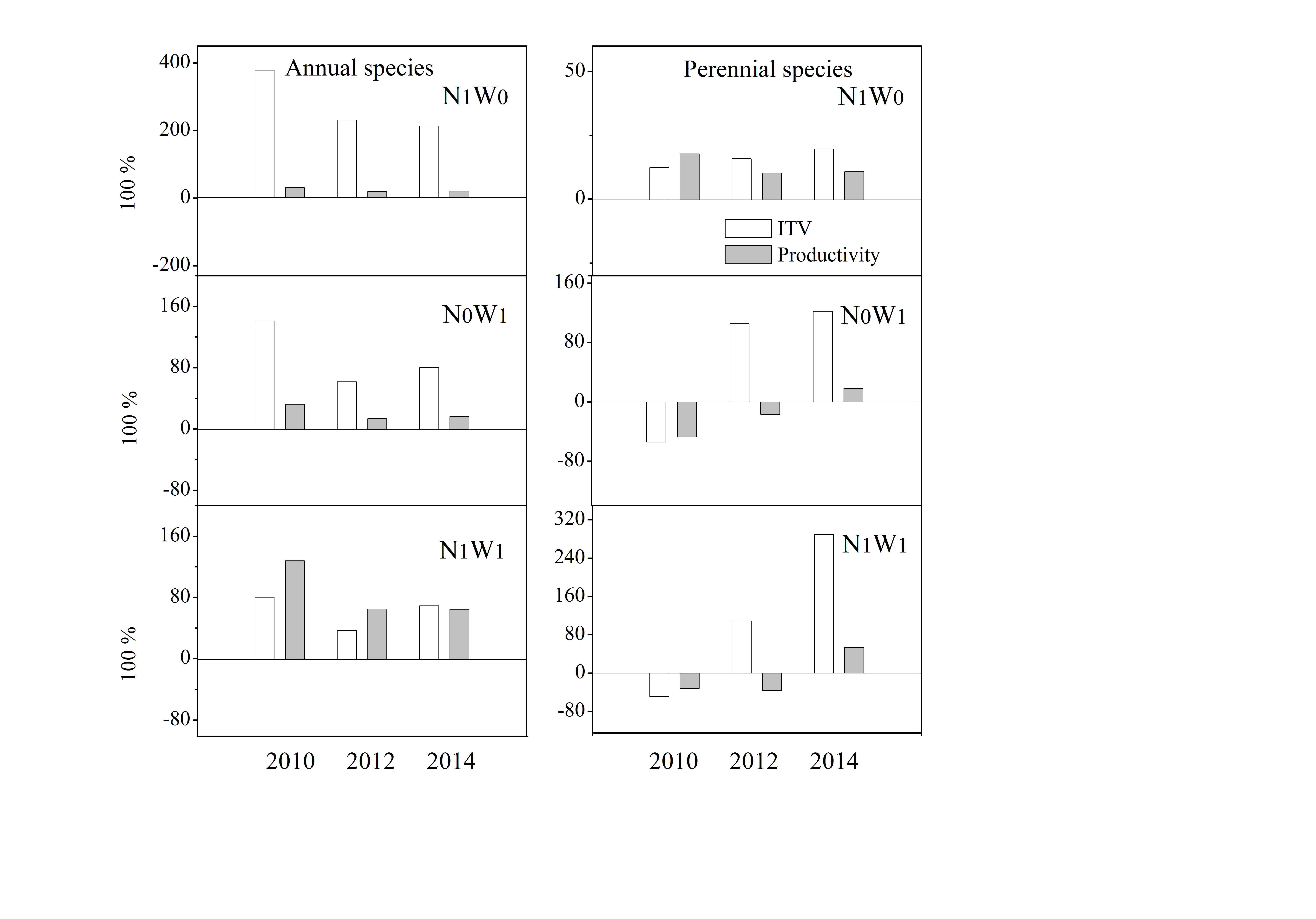

Supplement: Supplementary file 2 [file Image_2.jpeg]
